# Modeling Reveals the Dependence of Hippocampal Neurogenesis Radiosensitivity on Age and Strain of Rats

**DOI:** 10.3389/fnins.2018.00980

**Published:** 2018-12-20

**Authors:** Eliedonna Cacao, Sidath Kapukotuwa, Francis A. Cucinotta

**Affiliations:** Department of Health Physics and Diagnostic Sciences, University of Nevada, Las Vegas, NV, United States

**Keywords:** rat hippocampal neurogenesis, acute radiation exposure, fractionated radiation treatment, cancer radiotherapy, radiosensitivity of neurogenesis

## Abstract

Cognitive dysfunction following radiation treatment for brain cancers in both children and adults have been correlated to impairment of neurogenesis in the hippocampal dentate gyrus. Various species and strains of rodent models have been used to study radiation-induced changes in neurogenesis and these investigations have utilized only a limited number of doses, dose-fractions, age and time after exposures conditions. In this paper, we have extended our previous mathematical model of radiation-induced hippocampal neurogenesis impairment of C57BL/6 mice to delineate the time, age, and dose dependent alterations in neurogenesis of a diverse strain of rats. To the best of our knowledge, this is the first predictive mathematical model to be published about hippocampal neurogenesis impairment for a variety of rat strains after acute or fractionated exposures to low linear energy transfer (low LET) radiation, such as X-rays and γ-rays, which are conventionally used in cancer radiation therapy. We considered four compartments to model hippocampal neurogenesis and its impairment following radiation exposures. Compartments include: (1) neural stem cells (NSCs), (2) neuronal progenitor cells or neuroblasts (NB), (3) immature neurons (ImN), and (4) glioblasts (GB). Additional consideration of dose and time after irradiation dependence of microglial activation and a possible shift of NSC proliferation from neurogenesis to gliogenesis at higher doses is established. Using a system of non-linear ordinary differential equations (ODEs), characterization of rat strain and age-related dynamics of hippocampal neurogenesis for unirradiated and irradiated conditions is developed. The model is augmented with the description of feedback regulation on early and late neuronal proliferation following radiation exposure. Predictions for dose-fraction regimes compared to acute radiation exposures, along with the dependence of neurogenesis sensitivity to radiation on age and strain of rats are discussed. A major result of this work is predictions of the rat strain and age dependent differences in radiation sensitivity and sub-lethal damage repair that can be used for predictions for arbitrary dose and dose-fractionation schedules.

## Introduction

Since the first report of adult mammalian neurogenesis by Altman (1962), there has been many significant advancements and progress in studying neurogenesis, more particularly its response to different external stressors, one of which is radiation. Ionizing radiation can significantly impact neurogenesis in the hippocampus and negatively affect its function such as learning and cognition. Majority of patients (about 50–90%) who have undergone radiation treatment for primary and metastatic brain cancer exhibit cognitive dysfunctions that greatly affect the patient's quality of life (Makale et al., 2017). Several studies have examined the neurobiological response of radiation-induced brain injury and its correlation to cognition (Kim et al., 2013; Yang et al., 2017). However, mechanistic understanding of radiation-induced cognitive decline is poorly defined, perhaps due to its complexity that involves several interacting and synergistic factors, such as vascular damage, neuroinflammation, neurogenesis, and alterations to central nervous system (CNS) microenvironment (Greene-Schloesser et al., 2012; Makale et al., 2017).

Continuously producing new neurons throughout life, neurogenesis has been persistently detected in two regions of the adult brain: subventricular zone (SVZ) lining the lateral ventricular and subgranural zone (SGZ) of the hippocampus (Deng et al., 2010). Although recently, the presence of neurogenic precursor cells in the adult basolateral amygdala of adult mice was observed that generated functional interneurons (Jhaveri et al., 2018). The functional role of neurogenesis in the hippocampal dentate gyrus is still not well-defined but is reported to have an important role in learning and memory (Kempermann et al., 2004; Deng et al., 2010). Various precursor cell populations in the hippocampus have been described to produce new neurons, including astrocytes, oligodendrocytes and other glial cells (Doetsch, 2003; Seri et al., 2004; Steiner et al., 2004; Bonaguidi et al., 2011; Encinas et al., 2011; Kempermann et al., 2015). Lineage tracing of newly born neurons have been investigated to discern properties of neural stem cells (NSC) and their cell fate. However, different conclusions have been drawn from these studies that may in part be due to various labeling approaches of cell populations (Bonaguidi et al., 2012). Still, a unified hypothesis has been developed suggesting that NSC in the hippocampus undergo maintenance via self-renewal and generate new neurons, astrocytes and other glial cells (Alvarez-Buylla et al., 2001; Bonaguidi et al., 2012).

Investigations about hippocampal neurogenesis have used a variety of animal species (Lazic, 2012), although rodent models are extensively utilized. Evaluation of the differences in cell proliferation, neuroblasts differentiation and integration into mature granule cells in nine strains of mouse suggested neurogenesis is more prominent in C57BL/6 and ICR strains (Kempermann et al., 1997; Kim et al., 2017). Studies in rat hippocampal neurogenesis have seen differences in proliferation and survival of neuronal stem and progenitor cells between Sprague Dawley and spontaneously hypertensive rats (SHR) that may contribute to discrepancy in spatial memory functions (Perfilieva et al., 2001). Likewise, dissimilarity of neurogenesis between Sprague Dawley and Long Evans rats in response to spatial learning may alter rates of neuron cell maturation (Epp et al., 2011). Genetic background, environmental factors and hormonal stimuli are cited to contribute in several possible mechanisms that could account for observed strain-related differences in neurogenesis (Boss et al., 1985; Johnson and Mitchell, 2003; Kim et al., 2017).

Published studies in rodent models have shown that hippocampal neurogenesis is altered after exposures to low linear energy transfer (low LET) radiation, such as X-rays and γ-rays that are conventionally used in cancer radiation therapy (Peibner et al., 1999; Tada et al., 2000; Monje et al., 2002, 2003; Mizumatsu et al., 2003; Rola et al., 2004; Fukuda et al., 2005; Otsuka et al., 2006; Schindler et al., 2008; Achanta et al., 2009; Kalm et al., 2009; Conner et al., 2010; Tan et al., 2011; Jenrow et al., 2013; Blomstrand et al., 2014; Greene-Schloesser et al., 2014). However, these investigations have been limited in number of doses, post-irradiation time points and age at irradiation. Moreover, these studies have utilized different species and strains of rodent models despite the reported strain-dependence of hippocampal neurogenesis. Thus, mathematical modeling can serve as a useful tool to interpret experimental results and extrapolate to other conditions. We have previously developed a model of neurogenesis impairment after radiation exposure in C57BL/6 mouse (Cacao and Cucinotta, 2016a,b). The main purpose of this paper is to develop a more global model of radiation-induced alterations in rodent hippocampal neurogenesis by extending our earlier model to accommodate data in rat experimentation. Our model uses a system of non-linear differential equations (ODEs) to represent age, time after irradiation and dose-dependent changes to major neuronal cell population participating in neurogenesis that are reported in rodent experiments. Extensive comparison to experimental data and several predictions of the model including the dependence of radiosensitivity on age and strain of rats are discussed.

## Methods

### Adult Hippocampal Neurogenesis Model: Unperturbed Condition

**Figure 1:**
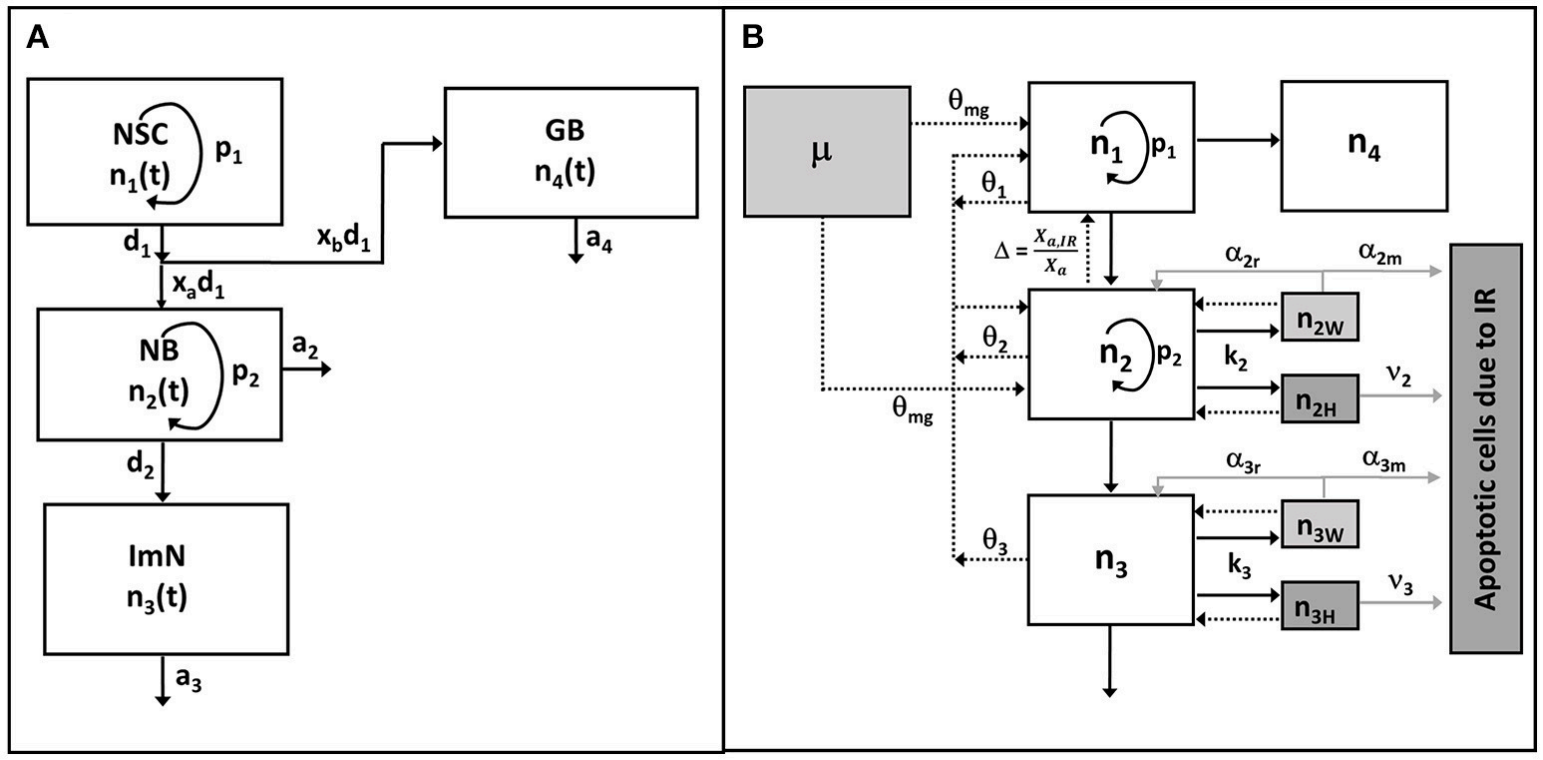
Schematic diagram of hippocampal neurogenesis model. **(A)** In unperturbed (control or sham) condition, neuronal cell population n_1_, n_2_, n_3_, n_4_ represent neural stem cell (NSC), amplifying neuronal progenitor cell or neuroblast (NB), immature neuron (Imn), and glioblast (GB), respectively. **(B)** After exposures to radiation, cells are classified as undamaged (n_i_), weakly damaged (n_iw_), and heavily damaged (n_iH_), where subscript *i* denotes type of neuronal cell population. Radiation damages are described by rate constants k_2_, k_3_ (assuming NSC and GB are radioresistant) while damage repair rates are depicted by α_2r_, α_3r._ Number of apoptotic cells due to radiation damages are defined from rate of apoptosis of heavily damaged cells ν_2_, ν_3_ and misrepaired weakly damaged cells α_2m_, α_3m_ (gray solid lines). Black solid arrows denote cellular differentiation or transfer at specific rates, while arrows with dashed lines represent the implicit feedback regulation upon the reproduction (p_1_) of NSC and proliferation (p_2_) of NB, with dissimilar contributions θ_1_, θ_2_, θ_3_, and θ_mg_ for NSC, NB, ImN, and activated microglia (μ), respectively. Feedback due to observed shift in neurogenic fate of newly born neurons is represented by Δ.

In this paper, we extend our previous model of mouse hippocampal neurogenesis to integrate the observed experimental data in rats and establish a more global model of rodent hippocampal neurogenesis. Briefly, we have considered four compartments that represent neuronal cell population, as illustrated in Figure 1A, to describe neurogenesis: (1) neural stem cells (NSC, n_1_), (2) amplifying neuronal progenitor cells or neuroblasts (NB, n_2_), (3) immature neurons (ImN, n_3_), (4) glioblasts (GB, n_4_). Based on several assumptions (Cacao and Cucinotta, 2016a), the dynamics of the unirradiated neuronal cell population can be described by the following non-linear ordinary differential equations:

(1) $$\begin{array}{r} \frac{dn_{1}\left(t\right)}{dt}=p_{1}n_{1}\left(t\right)-d_{1}n_{1}\left(t\right) \end{array}$$

(2) $$\begin{array}{r} \frac{dn_{2}\left(t\right)}{dt}=p_{2}x_{a}d_{1}\;n_{1}\left(t\right)-d_{2}n_{2}\left(t\right)\;-a_{2}n_{2}\left(t\right) \end{array}$$

(3) $$\begin{array}{r} \frac{dn_{3}\left(t\right)}{dt}=d_{2}n_{2}\left(t\right)-a_{3}n_{3}\left(t\right) \end{array}$$

(4) $$\begin{array}{r} \frac{dn_{4}\left(t\right)}{dt}=x_{b}d_{1}n_{1}\left(t\right)-a_{4}n_{4}\left(t\right) \end{array}$$

where d_1_ and d_2_ are rates of differentiation from NSC to NB and NB to ImN, respectively; a_2_, a_3_, a_4_ are apoptosis rates of NB, ImN, and GB, respectively; x_a_ and x_b_ are the fraction of NSC that differentiate into NB and GB, respectively, such that x_a_ + x_b_ = 1; p_1_ is the rate of NSC proliferation; and p_2_ is a factor to represent the proliferation of neuronal progenitors. Similar to our previous model and other mathematical models of neurogenesis (Ziebell et al., 2014, 2018; Li et al., 2017), we have assumed that all cell types (n_2_, n_3_, n_4_) undergo decay through apoptosis except for neural stem cells (n_1_). Moreover, in our earlier model, we have set p_2_ as a constant, which is sufficient to describe neurogenesis in mouse models. However, to accommodate results from rat experiments where increased proliferation in the first week after radiation exposure is observed, we have used a proliferation expression that is based on Sminorva's model of feedback regulation by different cell populations (Smirnova, 2011; Smirnova et al., 2014a,b). Thus, NSC and NB proliferation can be expressed as:

(5) $$\begin{array}{r} \begin{array}{c} \;p_{j}^{\;}=\frac{{\text{Ψ}}_{j}}{1+\left(\theta_{1}n_{1}+\theta_{2}n_{2}+\theta_{3}n_{3}\right)} \end{array} \end{array}$$

where Ψ_j_ is the maximum proliferation rate for NSC (*j* = 1) or NB (*j* = 2) and multipliers θ_1_, θ_2_, θ_3_ represent the dissimilar contributions of NSC, NB, and ImN in the negative feedback on NSC and NB proliferation. Parameters for different rat strain are obtained based on experimental data as summarized on Supplementary Table 2.

### Dynamics of Neuronal Cell Population After Irradiation

Figure 1B shows a schematic diagram of the hippocampal neurogenesis model upon exposure to radiation where effects of radiation on neuronal cell populations are integrated into Equations (1)–(4). In addition, a cell population is added to describe the number of apoptotic cells due to irradiation (n_5_). After irradiation, we have classified radiosensitive cells as undamaged (n_i_), weakly damaged (n_iW_) and heavily damaged (n_iH_) based on their radiation response and extent of damage, where i = 1–4 which denotes the four neuronal cell populations being considered in the model. We assume that weakly damaged cells are partially repairable while heavily damaged cells, as well as misrepaired weakly damaged cells, both lead to apoptosis. Therefore, the dynamics of neuronal cell populations after irradiation can be described by the following 13 coupled ordinary differential equations:

(6) $$\begin{array}{r} \frac{dn_{1}\left(t\right)}{dt}=p_{1}n_{1}\left(t\right)-d_{1}n_{1}\left(t\right)-k_{1}n_{1}\left(t\right)+\alpha_{1r}n_{1w}\left(t\right) \end{array}$$

(6a) $$\begin{array}{r} \frac{dn_{1w}\left(t\right)}{dt}=k_{1w}n_{1}\left(t\right)-\alpha_{1}n_{1w}\left(t\right) \end{array}$$

(6b) $$\begin{array}{r} \frac{dn_{1H}\left(t\right)}{dt}=k_{1H}n_{2}\left(t\right)-\nu_{1}n_{1H}\left(t\right) \end{array}$$

(7) $$\begin{array}{l} \frac{dn_{2}\left(t\right)}{dt}=p_{2}x_{a}d_{1}\;n_{1}\left(t\right)-d_{2}n_{2}\left(t\right)\;-a_{2}n_{2}\left(t\right)-k_{2}n_{2}\left(t\right)\\ \;+\alpha_{2r}n_{2w}\left(t\right) \end{array}$$

(7a) $$\begin{array}{r} \frac{dn_{2w}\left(t\right)}{dt}=k_{2w}n_{2}\left(t\right)-\alpha_{2}n_{2w}\left(t\right) \end{array}$$

(7b) $$\begin{array}{r} \frac{dn_{2H}\left(t\right)}{dt}=k_{2H}n_{2}\left(t\right)-\nu_{2}n_{2H}\left(t\right) \end{array}$$

(8) $$\begin{array}{r} \frac{dn_{3}\left(t\right)}{dt}=d_{2}n_{2}\left(t\right)-a_{3}n_{3}\left(t\right)-k_{3}n_{3}\left(t\right)+\alpha_{3r}n_{3w}\left(t\right) \end{array}$$

(8a) $$\begin{array}{r} \frac{dn_{3w}\left(t\right)}{dt}=k_{3w}n_{3}\left(t\right)-\alpha_{3}n_{3w}\left(t\right) \end{array}$$

(8b) $$\begin{array}{r} \frac{dn_{3H}\left(t\right)}{dt}=k_{3H}n_{3}\left(t\right)-\nu_{3}n_{3H}\left(t\right) \end{array}$$

(9) $$\begin{array}{r} \frac{dn_{4}\left(t\right)}{dt}=x_{b}d_{1}n_{1}\left(t\right)-a_{4}n_{4}\left(t\right)-k_{4}n_{4}\left(t\right)+\alpha_{4r}n_{4w}\left(t\right) \end{array}$$

(9a) $$\begin{array}{r} \frac{dn_{4w}\left(t\right)}{dt}=k_{4w}n_{4}\left(t\right)-\alpha_{4}n_{4w}\left(t\right) \end{array}$$

(9b) $$\begin{array}{r} \frac{dn_{4H}\left(t\right)}{dt}=k_{4H}n_{4}\left(t\right)-\nu_{4}n_{4H}\left(t\right) \end{array}$$

(10) $$\begin{array}{r} \frac{dn_{5}\left(t\right)}{dt}=\alpha_{2m}n_{2w}\left(t\right)+\nu_{2}n_{2H}\left(t\right)+\alpha_{3m}n_{3w}\left(t\right)\\ \;+\nu_{3}n_{3H}\left(t\right)-\nu_{5}n_{5}\left(t\right) \end{array}$$

The rates of radiation damage and repair are described by k_i_ and α_i_, respectively. We assume that the repair rate is a fraction of damage rate defined by Equation (11). Radiation lesions are divided into two components to illustrate weakly (k_iW_) and heavily (k_iH_) damaged cells. A fraction of weakly damaged cells are successfully repaired with rate constant α_ir_, while misrepaired cells undergo apoptosis with rate constant α_im_. Thus, the weakly damage cells are a mathematical description of sub-lethal radiation damage. Finally, assuming ξ_i_ as the fraction of repairable weakly damaged cells, radiation damage and repair rates can be re-written as:

(11) $$\begin{array}{r} \alpha_{i}=\omega k_{i} \end{array}$$

(12) $$\begin{array}{r} \alpha_{i}=\alpha_{ir}+{\alpha_{i}}_{m} \end{array}$$

(13a) $$\begin{array}{r} \alpha_{ir}=\xi_{i}\alpha_{i} \end{array}$$

(13b) $$\begin{array}{r} \alpha_{im}=\left(1-\xi_{i}\right)\alpha_{i} \end{array}$$

Heavily damaged cells and misrepaired weakly damaged cells are assumed to undergo apoptosis at the rate of ν_i_ and α_im_, respectively. It should be noted that heavily damaged cells lead directly to apoptosis rapidly with no time delay assumed, while misrepaired weakly damaged cells undergo apoptosis after a time delay, such that ν_i_ > α_im_.

The NSC and NB proliferation rate described in Equation (5) is modified to account for radiation damages. Dimensionless multipliers Φ and Γ are included to represent the dissimilar contributions of weakly and heavily damaged cells due to differences in their specific death rates as described by Equation (14). Other factors that affect proliferation can be included in Equation (15).

(14) $$\begin{array}{r} \frac{\Gamma }{\Phi }=\frac{\nu_{i}}{\alpha_{im}} \end{array}$$

(15) $$\begin{array}{r} p_{j,IR}=\\ \frac{{\text{Ψ}}_{i}}{1+\theta_{1}n_{1}+\theta_{2}\left(n_{2}+\Phi n_{2W}+\Gamma n_{2H}\right)+\theta_{3}\left(n_{3}+\Phi n_{3W}+\Gamma n_{3H}\right)} \end{array}$$

Parametric forms to describe effects of activated microglial cells on proliferation and neurogenic cell fate of hippocampal neurogenesis are considered. Equations (16) and (17) are parametric equations to describe the fractional increase in activated microglia (μ) and decrease in neurogenic fate (Δ = x_a, IR_/x_a_) as a function of post-irradiation time (t_postIR_) and dose based on the rodent experiments as summarized in Supplementary Table 3. In some rat strain, it was observed that increased number of activated microglia and a decrease in neurogenic fate only starts at approximately 30 days post-irradiation time. Therefore, in some cases, Equations (16) and (17) are constrained and expressed in terms of a time delay, τ = t – t_d_:

(16) $$\frac{d\mu (\tau )}{dt}=\left\{ \begin{array}{l} 0\;for\;t<t_{d}\\ \left[A_{0}\left(\frac{dose}{dose+A_{1}}\right)+B\tau +C\tau^{2}\right]e^{-\lambda \tau }\;for\;t\geq t_{d} \end{array}\right.$$

(17) $$\frac{d\Delta (\tau )}{dt}=\left\{ \begin{array}{l} 0\;for\;t<t_{d}\\ \left[A_{0}\left(\frac{dose}{dose+A_{1}}\right)+B_{0}\mu +B_{1}\mu \tau +C\tau^{2}\right]e^{-\lambda \tau }\;for\;t\geq t_{d} \end{array}\right.$$

Taking into account the negative feedback caused by the increase in activated microglial cells, the proliferation rate can be re-written as:

(18) $$\begin{array}{r} \begin{array}{c} p_{j,IR}=\frac{{\text{Ψ}}_{j}}{1+\theta_{1}n_{1}+\theta_{2}\left(n_{2}+\Phi n_{2W}+\Gamma n_{2H}\right)+\theta_{3}\left(n_{3}+\Phi n_{3W}+\Gamma n_{3H}\right)+\theta_{mg}\mu } \end{array}\; \end{array}$$

where the coefficient θ_mg_ represents the contribution of increased number of activated microglia on proliferation.

A unified hypothesis of neurogenesis suggests that NSC in the hippocampus generate new neurons, astrocytes and other glial cells (Alvarez-Buylla et al., 2001; Bonaguidi et al., 2012). A non-neural fate for NSCs has not been identified (Bonaguidi et al., 2011; Pilz et al., 2018), however some studies have indicated a role for endothelial cells through neurogenic-angiogenic anatomical and signaling relationships (Palmer et al., 2000; Monje et al., 2002). We have not included endothelial cells in our model because studies have indicated that vascular damage in rats after radiation exposure only occurs at high doses (single dose of >20 Gy), which is more than the clinically relevant dose in humans (10 Gy dose in rats) (Calvo et al., 1988; Hodges et al., 1998), and that irradiation did not significantly alter the proportion of newborn endothelial cells (Monje et al., 2002) and the reduction of neuronal progenitor cells after irradiation was not due to vasculature damage (Otsuka et al., 2006). On the other hand, studies on low LET radiation effects on neurogenesis suggest that neuronal stem cells are resistant to radiation for doses up to about 10–20 Gy in rodent models (Fike et al., 2007; Andres-Mach et al., 2008; Rivera et al., 2013; DeCarolis et al., 2014). Furthermore, gliogenesis was found to be more radiation resistant than production of new neurons (Monje et al., 2002; Mizumatsu et al., 2003; Rola et al., 2004). Highly proliferating neuronal precursor cells and their progeny, immature neurons, are extremely sensitive to irradiation, undergoing apoptosis after clinically relevant doses, regardless of rodent species (mice or rats) (Fike et al., 2007). Therefore, we focus on the effects of radiation on neuroblasts and immature neurons that are extensively studied in rodent models, such that k_2_,k_3_ >> k_1_,k_4_. However, the dynamics of radiation damage and repair for NSC,GB and endothelial cells for higher radiation doses can be formulated for future studies or when experimental data have been made available.

### Acute Radiation Exposure

For an acute irradiation, it is plausible to assume that rates corresponding to proliferation and differentiation, as well as damage repair, are negligible compared to the rates for damage induction during irradiation period (a few minutes or less). This leads to a simplified version of the model that can be readily integrated, with the following solutions at the end of acute exposure of duration (t_IR_):

(19) $$\begin{array}{r} n_{i}\left(t_{IR}\right)=n_{i}\left(0\right)\;e^{\left(-k_{i}t_{IR}\right)}=n_{i}\left(0\right)\;e^{\left(-\frac{D}{D_{0i}}\right)} \end{array}$$

(20) $$\begin{array}{r} n_{iW}\left(t_{IR}\right)=n_{i}\left(0\right)\left(\frac{D_{0i}}{D_{0iW}}\right)\;\left[1-e^{\left(-\frac{D}{D_{0i}}\right)}\right] \end{array}$$

(21) $$\begin{array}{r} n_{iH}\left(t_{IR}\right)=n_{i}\left(0\right)\left(\frac{D_{0i}}{D_{0iH}}\right)\;\left[1-e^{\left(-\frac{D}{D_{0i}}\right)}\right] \end{array}$$

The terms k_i_t_IR_, k_iW_t_IR_, and k_iH_t_IR_ for acute irradiation are conveniently re-expressed as D/D_0i_, D/D_0iW_, and D/D_0iH_, respectively, where D is the absorbed dose in Gy, D_0i_, D_0iW_, D_0iH_ are the characteristic doses where 37% of the cells are undamaged, weakly damaged, and heavily damaged, respectively, where the D_0i_ term obey:

(22) $$\begin{array}{r} \frac{1}{D_{0i}}=\frac{1}{D_{0iW}}+\frac{1}{D_{0iH}} \end{array}$$

Equations (19)–(21) then become the initial conditions to solve Equations (6)–(10) with the k_i_-terms no longer contributing for times after irradiation is discontinued.

### Fractionated Irradiation

Equations (6)–(10) can be integrated directly for a chronic exposure. However, if fractionated radiation exposure is administered, the equations are solved in two-steps for each radiation fraction first with the k-terms on and followed by a second time-period with the k-terms off.

### Data Analysis and Mathematical Modeling

All data analysis and modeling are accomplished using Matlab 2015a (Mathworks, Inc.). Fitting is done using the built-in curve fitting tool. Solver function ode45 is used to solve the system of ordinary differential equations describing the dynamics of un-irradiated and irradiated neurogenesis.

## Results

### Age and Strain Dependence of NSC and Their Progeny in Unirradiated Rats

**Figure 2:**
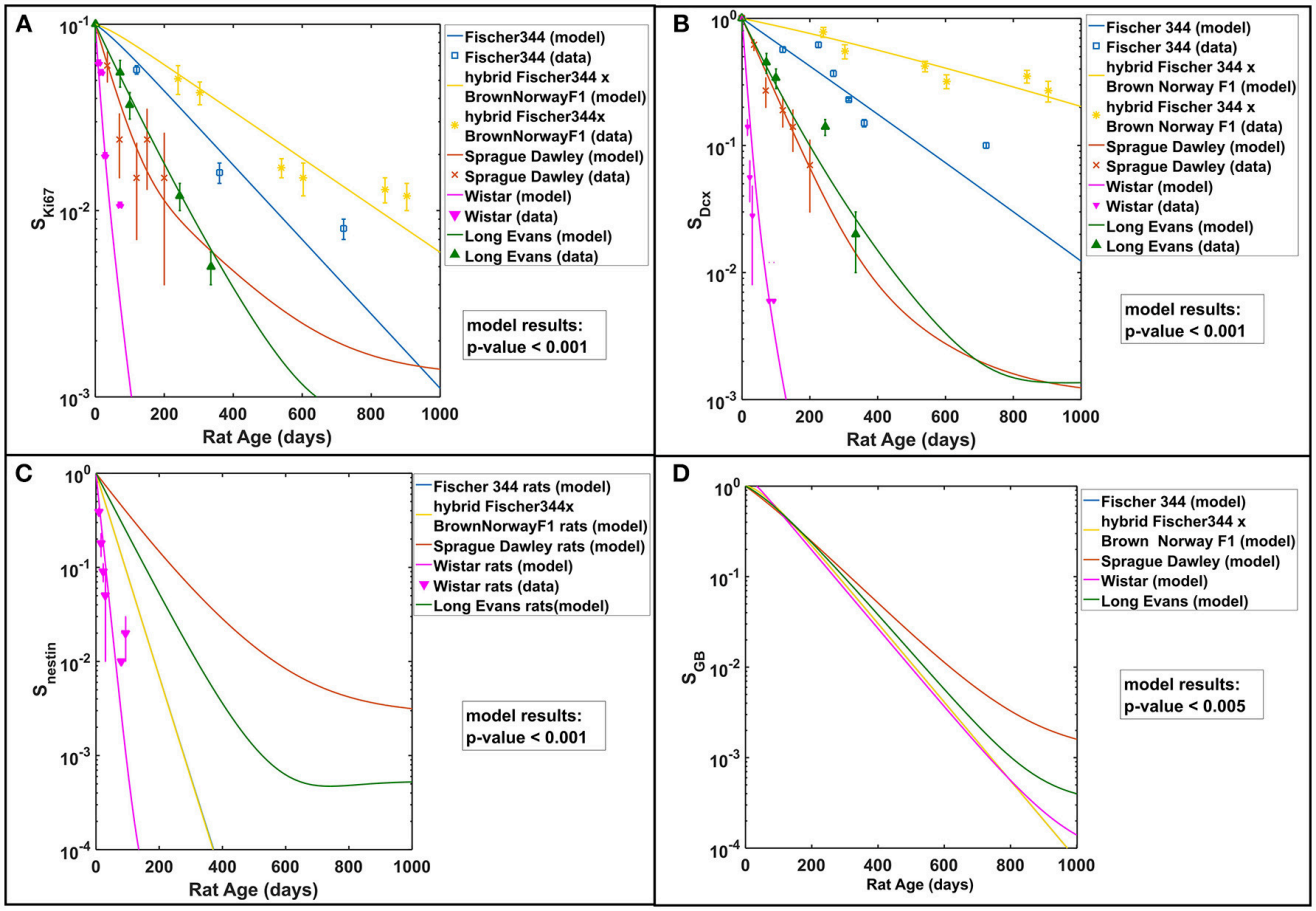
Rat strain and age dependence of hippocampal neurogenesis. Modeling results in comparison with available experimental data of **(A)** neuronal progenitor cells or neuroblasts (NB) using Ki67 marker, **(B)** immature neurons (ImN) using Dcx marker, **(C)** neural stem cells (NSC) using nestin marker, and **(D)** glioblasts (GB). All cell populations are normalized against their respective initial values and described as surviving fraction (S_i_). Test of significance (*p*-values) among modeling results of different rat strains is done using one-way ANOVA.

Studies in rodent models have shown the age-related dynamics of hippocampal neurogenesis (Boss et al., 1985; Kuhn et al., 1996; Ben Abdallah et al., 2010; Walter et al., 2011; Kim et al., 2013; Beccari et al., 2017; Yang et al., 2017; Ziebell et al., 2018). We have previously developed a mathematical model based on standard approach of cell kinetics using systems of non-linear ordinary differential equations (ODEs) to analyze hippocampal neurogenesis in C57BL/6 mouse model (Cacao and Cucinotta, 2016a). In our earlier model, we considered four compartments that represent key cell populations in neurogenesis: neural stem cells (NSC), neuronal progenitor cells or neuroblasts (NB), immature neurons (ImN), and glioblasts (GB). These cell populations are identified experimentally using markers such as nestin for NSC, Ki-67 for NB and doublecortin (Dcx) for ImN. As illustrated in the schematic diagram in Figure 1A, we assumed that neural stem cells are regulated by self-renewal (p_1_) and differentiate (d_1_) into neuronal progenitor cells and glial progenitor cells, with x_a_ and x_b_ representing fraction that differentiate into NB and GB, respectively. Then, neuronal progenitor cells proliferate (p_2_), expressed by an equation similar to NSC renewal (p_1_), and differentiate (d_2_) to form immature neurons. Finally, we have considered loss by apoptosis for NB, ImN and GB represented by a_2_, a_3_, and a_4_, respectively. Fractions of NSC that differentiate into neurons and glia are set equal to 0.60 and 0.40, respectively (Perfilieva et al., 2001; Monje et al., 2002; Greene-Schloesser et al., 2014). Initial cell populations used in the model are n_1_(0) = 8.6 × 10^4^, n_2_(0) = 7.3 × 10^4^, n_3_(0) = 2.9 × 10^5^, n_4_(0) = 8.8 × 10^4^ for NSC, NB, ImN, and GB, respectively. The NSC initial cell population is based on experimentally determined nestin ratio from rat whole-brain (RWB) and mouse whole-brain (MWB) derived stem cells of 1.8 (nestin_RWB_/nestin_MWB_ = 1.8) (Ray and Gage, 2006). The initial value of NB is determined by taking into consideration that granule cell proliferation in rats and C57BL/6 mice is similar (Snyder et al., 2009), therefore, we have used mouse NSC/NB = 1.2 at age = 0 (birth) derived from the ratio of NSC over amplifying neuroprogenitor (ANP) equal to 2.7 and ANP average division of 2.3 (Encinas et al., 2011). Subsequently, initial cell population values of ImN and GB are determined by using the ratio of NB over ImN (Amrein et al., 2011; Lazic, 2012) and ImN over GB (Verkhratsky and Butt, 2013; Ziebell et al., 2014), which are 0.25 and 3.3, respectively. Table 1 shows the summary of rat strain dependent parameters for hippocampal neurogenesis, while other parameters that do not depend on rat strain are presented in Supplementary Table 1. These parameters are estimated based on our model assumptions and available experimental data that are summarized in Supplementary Table 2. With these estimated parameters, the age-dependent neuronal cell population dynamics of hippocampal neurogenesis in different rat strains are established and presented in Figure 2. We compare to data for male rats in this paper as there was insufficient data in female rats across the different strains considered.

**Table 1:** Rat strain dependent parameters for hippocampal neurogenesis.

**Parameters (unit)**	**Fischer 344**	**Hybrid Fischer 344 × Brown Norway F1**	**Sprague Dawley**	**Wistar**	**Long Evans**
d_1_ (day^−1^)	2.5 × 10^−2^	2.5 × 10^−2^	1 × 10^−2^	7.5 × 10^−2^	1.5 × 10^−2^
d_2_ (day^−1^)	4.5 × 10^−3^	2.8 × 10^−3^	5 × 10^−3^	6 × 10^−2^	1 × 10^−3^
a_2_ (day^−1^)	1 × 10^−4^	1 × 10^−4^	1 × 10^−2^	8 × 10^−3^	1 × 10^−4^
a_3_ (day^−1^)	5.5 × 10^−3^	2 × 10^−3^	1.5 × 10^−2^	1 × 10^−1^	1.5 × 10^−2^

### Radiation Damage and Neurogenesis Impairment

A summary of experimental data describing the alterations of hippocampal neurogenesis in different rat strains caused by radiation exposures is presented in Supplementary Table 3. As shown in Figure 1B, these radiation-induced changes in neurogenesis are described in our model through radiation damage rate constants (k_i_) for neuronal cell populations, with kinetic rates of repair of weakly damaged cells (α_ir_) and apoptotic conversions of misrepaired weakly (α_im_) and heavily (ν_i_) damaged cells. For acute radiation exposures, radiation damage rate constants are expressed as characteristic dose parameters (D_0ij_) where subscripts i and j denotes neuronal cell population (i = 2 for NB and i = 3 for Imn) and degree of damage (j = W for weakly damaged and j = H for heavily damaged), respectively. These characteristic dose parameters represent the radiation dose (in Gy) where 37% of cells are either undamaged (D_0i_), weakly damaged (D_01W_), or heavily damaged (D_01H_). For instance, using Equation (19), when radiation dose (D) is equal to characteristic dose (D_0i_), the ratio of cell population after and before irradiation, n_i_(t_IR_)/n_i_(0), is 0.37, which indicates that 37% of the cell population is undamaged by radiation exposure. Table 2 shows the estimated radiation damage and repair related model parameters. Characteristic dose parameters vary with different rat strains and indicate that neuronal progenitor cells or neuroblasts (NB) are more sensitive to radiation treatment than immature neurons (ImN), except for Sprague Dawley rats where D_02_ and D_03_ are the same. Some parameters are chosen to have the same values as in our previous model for C57BL/6 mouse in order to minimize the number of parameters to be evaluated. On the other hand, no analysis or investigation has been carried out to provide a more precise estimate of other damage and repair related parameters such as fraction of weakly damaged cells (D_0i_/D_0iW_) and fraction of repairable weakly damaged cells (ξ_i_). Accordingly, we have analyzed variations in the aforementioned parameters and compare modeling results with experimental data, which gives rise to rat strain-dependence of characteristic doses and fraction of weakly and heavily damaged cells (D_0i_, D_0i_/D_0iW_, D_0i_/D_0iH_) and age-dependence of fraction of repairable weakly damaged cells (ξ_i_).

**Table 2:** Radiation damage and repair related model parameters.

**Parameters (unit)**	**Fischer 344**					**Hybrid Fischer 344 × Brown Norway F1**			**Sprague Dawley**				**Wistar**		
**CHARACTERISTIC DOSE PARAMETERS**															
D_02_ (Gy)	0.50					1.0			1.8				———		
D_02W_ (Gy)	0.71					2.0			2.25				———		
D_02H_ (Gy)	1.7					2.0			9				———		
D_03_ (Gy)	4.5					10			1.8				3		
D_03W_ (Gy)	22.5					12.5			180				15		
D_03H_ (Gy)	5.6					50			1.82				3.75		
**REPAIR RELATED PARAMETERS**															
Age (day)	63	77	84	98	109	240	540	840	21	50	70	84	9	23	180
ξ_2_	0.10	0.15	0.30	0.20	0.40	0.40	0.60	0.90	0.1	0.3	0.5	0.95	———		
ξ_3_	0.10					0.50	0.80	0.99	0.99				0.10	0.20	0.95

*−−−, No experimental dataavailable*.

**Figure 3:**
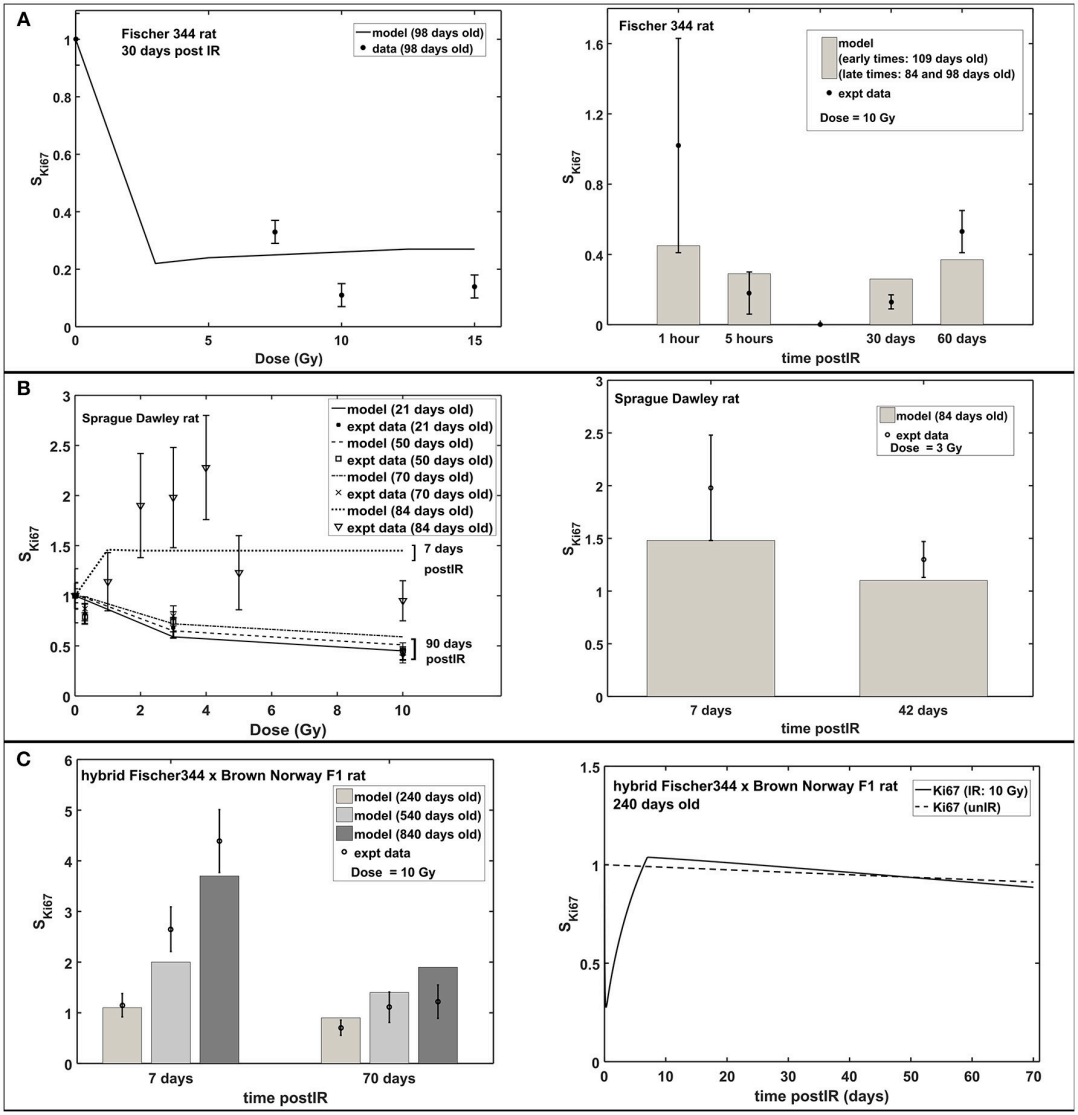
Response of proliferation marker Ki67 after acute radiation exposure: comparison between modeling results and experimental data. **(A)** Radiation dose and post-irradiation time response in Fischer 344 rat. **(B)** Radiation dose and post-irradiation time response in Sprague Dawley rat. **(C)** Post-irradiation time-dependent response in hybrid Fischer 344 × Brown Norway F1 rat. All cell populations are normalized against their respective age-matched control/unirradiated rat model and described as *S*_*Ki*67_.

**Figure 4:**
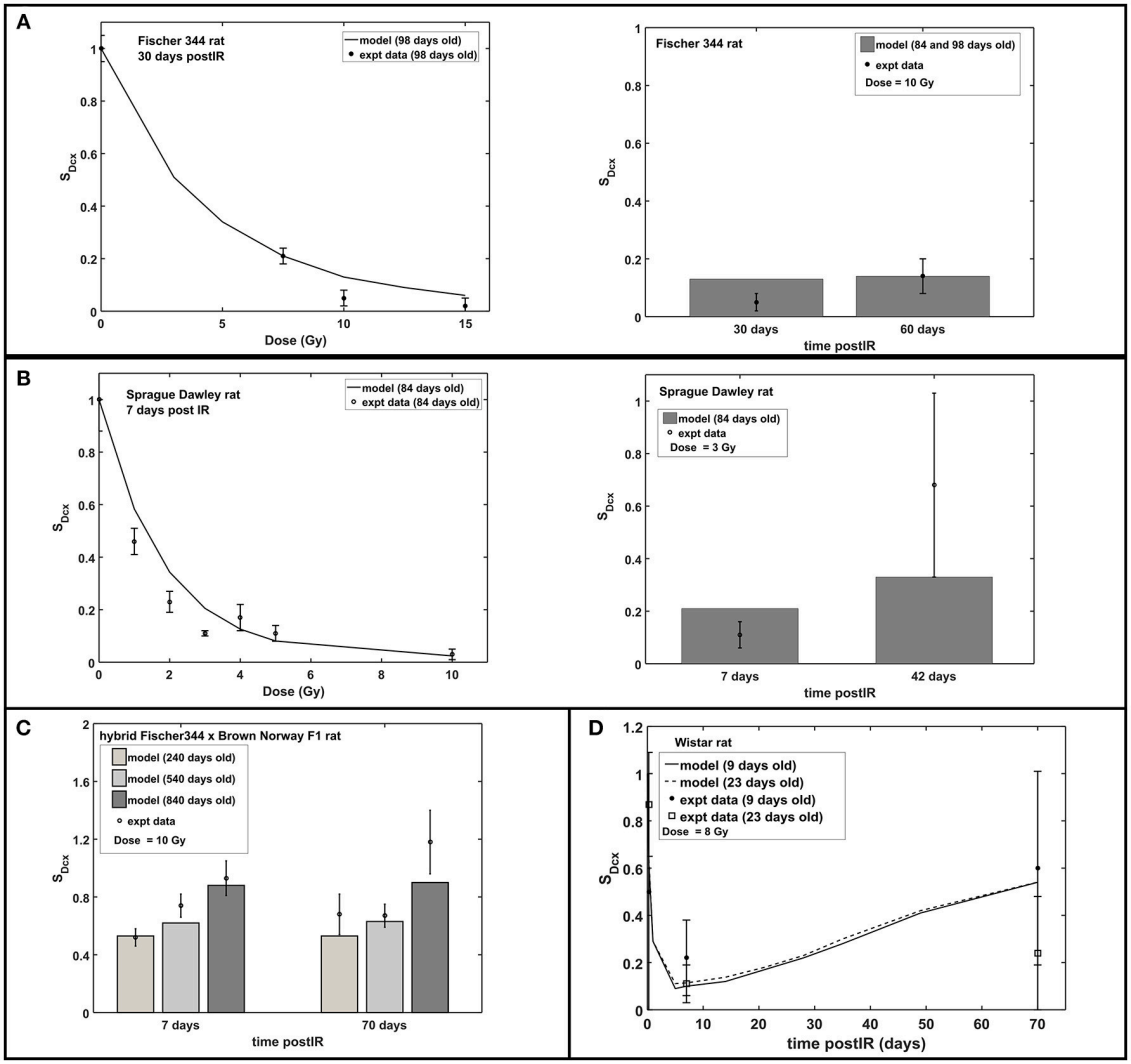
Response of immature neuron marker Dcx after acute radiation exposure: comparison between modeling results and experimental data. **(A)** Radiation dose and post-irradiation time response in Fischer 344 rat. **(B)** Radiation dose and post-irradiation time response in Sprague Dawley rat. **(C)** Post-irradiation time-dependent response in hybrid Fischer 344 × Brown Norway F1 rat. **(D)** Post-irradiation time-dependent response in Wistar rat. All cell populations are normalized against their respective age-matched control/unirradiated rat model and described as *S*_*Dcx*_.

Figure 3 shows the radiation dose and post-irradiation time response of proliferation marker Ki-67 after exposure to acute radiation for Fischer 344, Sprague Dawley and hybrid Fischer 344 × Brown Norway F1 rats. Modeling results have emulated the observed experimental data where a transient decrease in Ki67 occurs at 1–2 days post-irradiation, followed by an increase in proliferation that peaks at 7 days after radiation exposure then a steady decrease in proliferation at late post-irradiation times or months after exposure (see right panel in Figure 3C). On the contrary, as presented in Figure 4, the response in the immature neuron marker Dcx shows a dose-dependent decrease until 7 days post-irradiation then steadily increase at late post-irradiation times.

### Early and Late Response of Hippocampal Neurogenesis Upon Radiation Exposure

**Figure 5:**
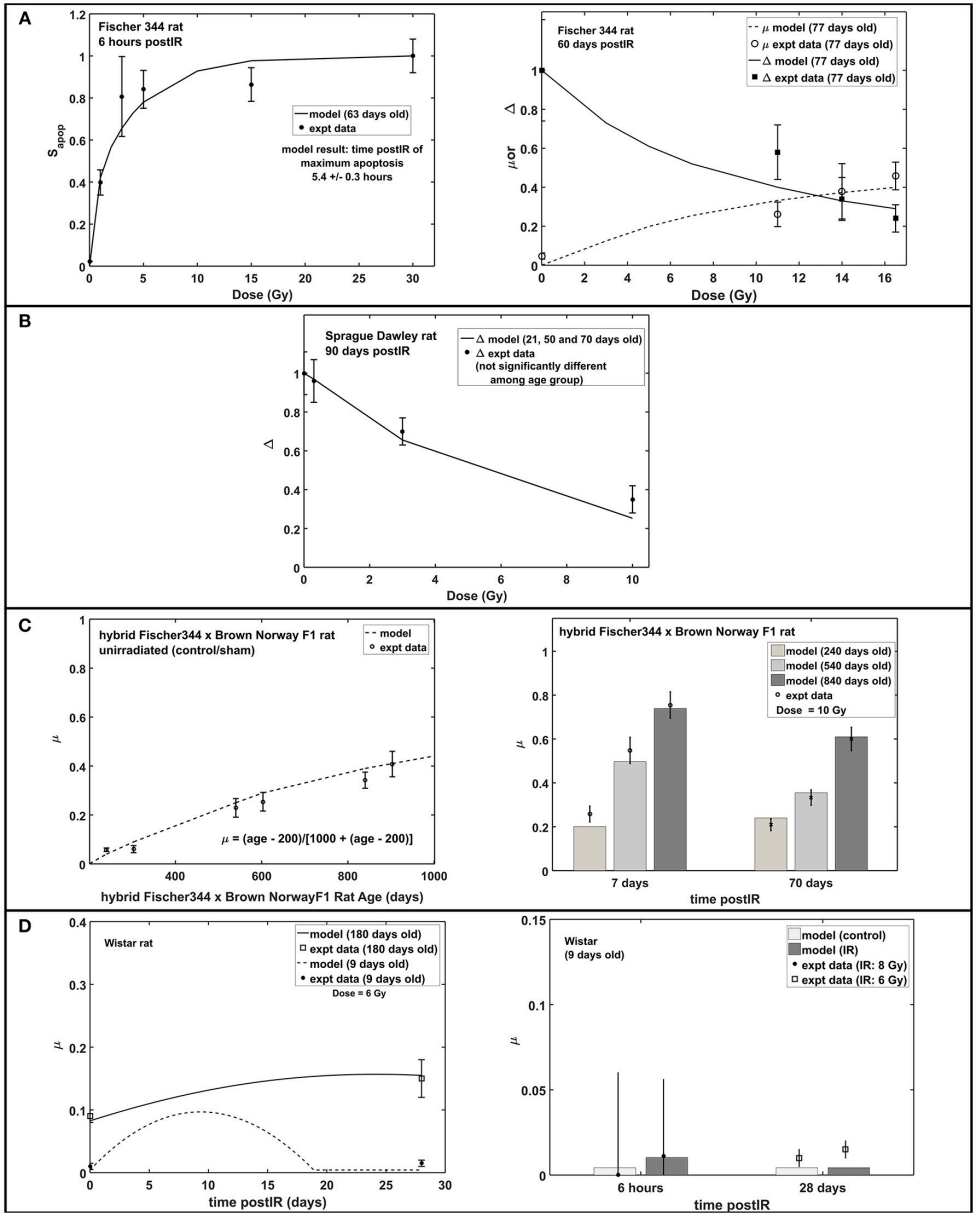
Apoptosis, microglial activation (μ) and neurogenic fate (Δ): early and late response of hippocampal neurogenesis after acute radiation exposure. **(A)** Dose-dependent response of apoptosis, microglial activation and neurogenic fate in Fischer 344 rat. **(B)** Dose-dependent response of microglial activation in Sprague Dawley rat. **(C)** Age-dependent microglial activation in unirradiated old age and post-irradiation time-dependent response in hybrid Fischer 344 × Brown Norway F1 rat. **(D)** Post-irradiation time-dependent response of microglial activation in Wistar rat. Apoptosis is expressed as fraction of maximum apoptosis and described as S_apop_.

Increased apoptosis, a short term effect of acute radiation exposure, is one of the causes of transient decrease in neuroblasts and immature neurons. In mouse models, apoptosis consists of a two-part response where a steeper slope at low doses represents significant loss of NB and a shallower slope at higher doses indicates ImN loss (Mizumatsu et al., 2003). Modeling and experimental results both exhibit the same two-part response of apoptosis in rats. As shown in Figure 5A (left panel), a sharp slope is recognized at doses <2 Gy and a moderate slope at doses 2–10 Gy. This two-part apoptosis response coincides with the estimated characteristic doses for NB (D_02_) and ImN (D_03_) for different rat strains (refer to Table 2), which ranges from 0 to 2 Gy for D_02_ and from 2 to 10 Gy for D_03_. Moreover, modeling results indicate maximum apoptosis occurs at 5.4 ± 0.3 h that is comparable to the observed experimental maximum apoptosis at 6 h (Tada et al., 2000).

Studies have shown that central nervous system (CNS) injury, such as brain ischemia (Tobin et al., 2014) and traumatic brain injury (Cho and Yun Kim, 2010), caused an increase in proliferation of neuronal progenitor cells in the hippocampus that occurs within 7 days after brain insults then quickly returns to baseline. Increased cell proliferation is also observed between 1 and 7 days after radiation exposure (Fike et al., 2007). Table 3 shows the estimated model parameters to describe the short term increase in NB proliferation, as well as the late response of hippocampal neurogenesis after radiation treatment that is characterized by increased microglial activation. Another late response after irradiation, a possible shift in neurogenic fate is described by the model. However, we found that adopting the neurogenic fate (Δ) parameters similar to our earlier model for mouse, as shown in Supplementary Table 1, are sufficient to emulate the observed dose-dependent data in Fischer 344 and Sprague Dawley rats, as presented in Figures 5A,B. Moreover, increased activation of microglial cells in irradiated and unirradiated (control/sham) hybrid Fischer 344 × Brown Norway F1 rats with aging and the greater increased and extended microglial activation in older Wistar rats are shown in Figures 5C,D, respectively.

**Table 3:** Model parameters to describe early and late radiation response of hippocampal neurogenesis.

**Parameters (unit)**	**Fischer 344**	**Hybrid Fischer 344 x Brown Norway F1**			**Sprague Dawley**	**Wistar**	
**NB PROLIFERATION PARAMETER**							
Ψ_2_ early response: t_postIR_ ≤ 7d	5 × 10^2^				6 × 10^4^	5 × 10^2^	
		$\{\begin{array}{l} 3\times 10^{4}\;for\;age\;=240\;day\;\\ 9\times 10^{4}\;for\;age\;=540\;day\;\\ 3\times 10^{5}\;for\;age\;=840\;days \end{array}$		
**ACTIVATED MICROGLIA (μ) RELATED PARAMETERS**							
Age (day)	63–109	240	540	840	21–84	9–23	180
t_d_ (days)	30	0			30	0	
A_0_	3.5 × 10^−2^	6 × 10^−2^	8 × 10^−2^	1.05 × 10^−1^	3.5 × 10^−2^	5 × 10^−2^	1.5 × 10^−2^
A_1_ (Gy)	9	9			9	9	9
B (day^−1^)	−7.5 × 10^−6^	−1 × 10^−3^			−7.5 × 10^−6^	−2 × 10^−3^	−7.5 × 10^−6^
C (day^−2^)	−1 × 10^−5^	−1.5 × 10^−5^			−1 × 10^−5^	−1.5 × 10^−5^	−1 × 10^−5^
λ (day^−1^)	3 × 10^−2^	6 × 10^−2^	2.5 × 10^−2^	1 × 10^−2^	3 × 10^−2^	1 × 10^−2^	3 × 10^−2^

### Altered Neurogenesis After Fractionated vs. Acute Exposures

**Figure 6:**
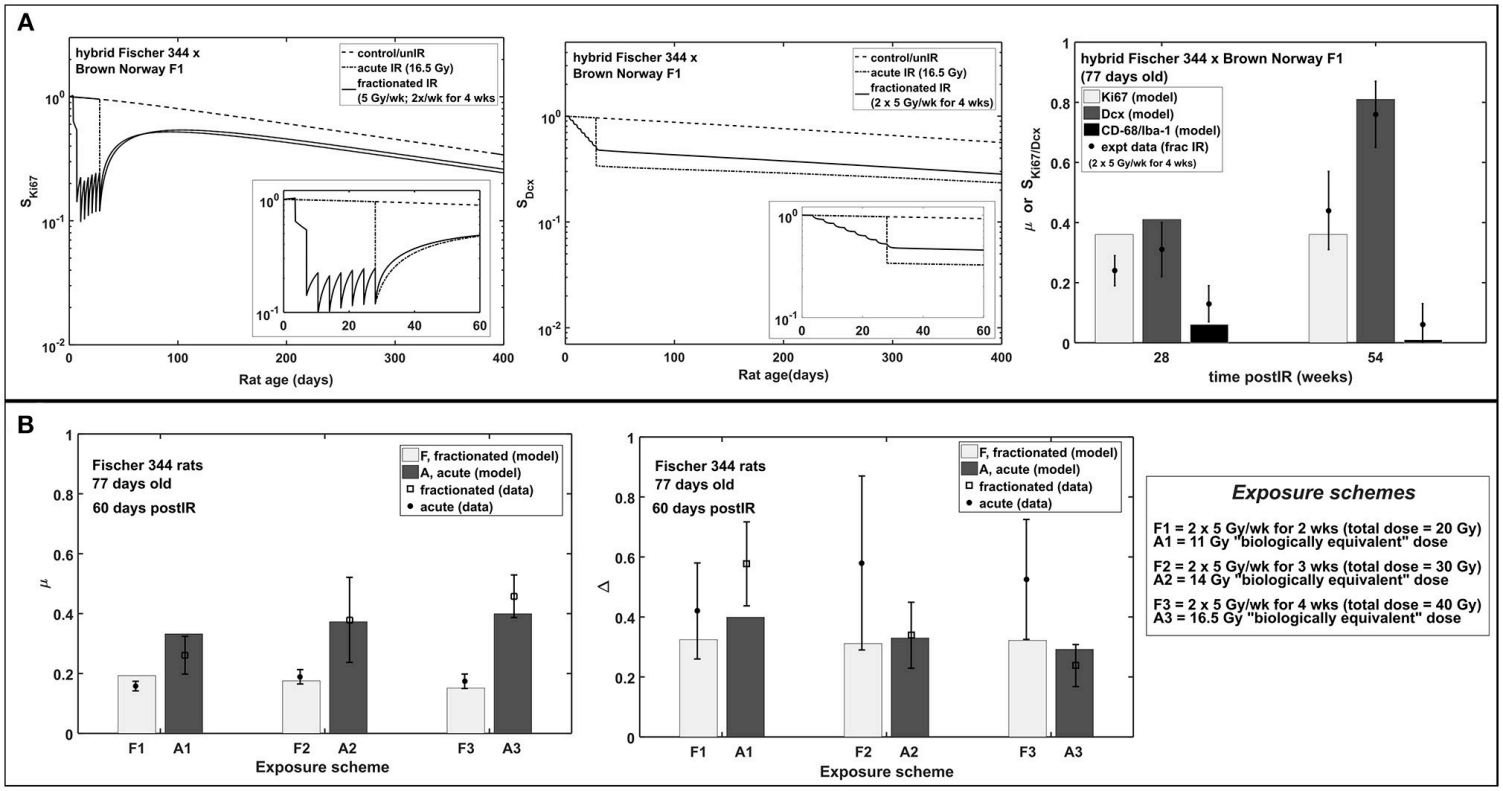
Acute vs. fractionated radiation exposure: modeling results compared with experimental data. **(A)** Modeling dynamics of acute and fractionated irradiation and response of proliferating marker Ki67, immature neuron marker Dcx, and fraction of activated microglia marker CD-68/Iba-1 in hybrid Fischer 344 × Brown Norway F1 rat. **(B)** Dose-dependent response of fraction of activated microglia (μ) and neurogenic fate (Δ) in Fischer 344 rat.

Figure 6 shows neurogenesis response to fractionated compared to acute radiation exposures in hybrid Fischer 344 x Brown Norway and Fischer 344 rats. In all these cases, acute radiation exposures refer to the “biologically equivalent” single doses (Greene-Schloesser et al., 2014) and are assumed to be applied on the final day of the fractionated schedule as illustrated in Figure 6A. Our modeling results favorably depict the observed neurogenesis response to fractionated radiation exposures in rat experiments. Modeling dynamics of proliferation marker Ki67 and immature neuron marker Dcx after acute and fractionated exposures compared to unirradiated control reveal that acute radiation exposure results in more damage than fractionated exposures in both short and long term effects. As shown in Figure 6B, at 60 days post-irradiation, increased activation of microglial cells is greater in acute than in fractionated radiation exposures. Meanwhile, a shift toward gliogenesis is favored by acute exposures compared to fractionated treatment.

**Figure 7:**
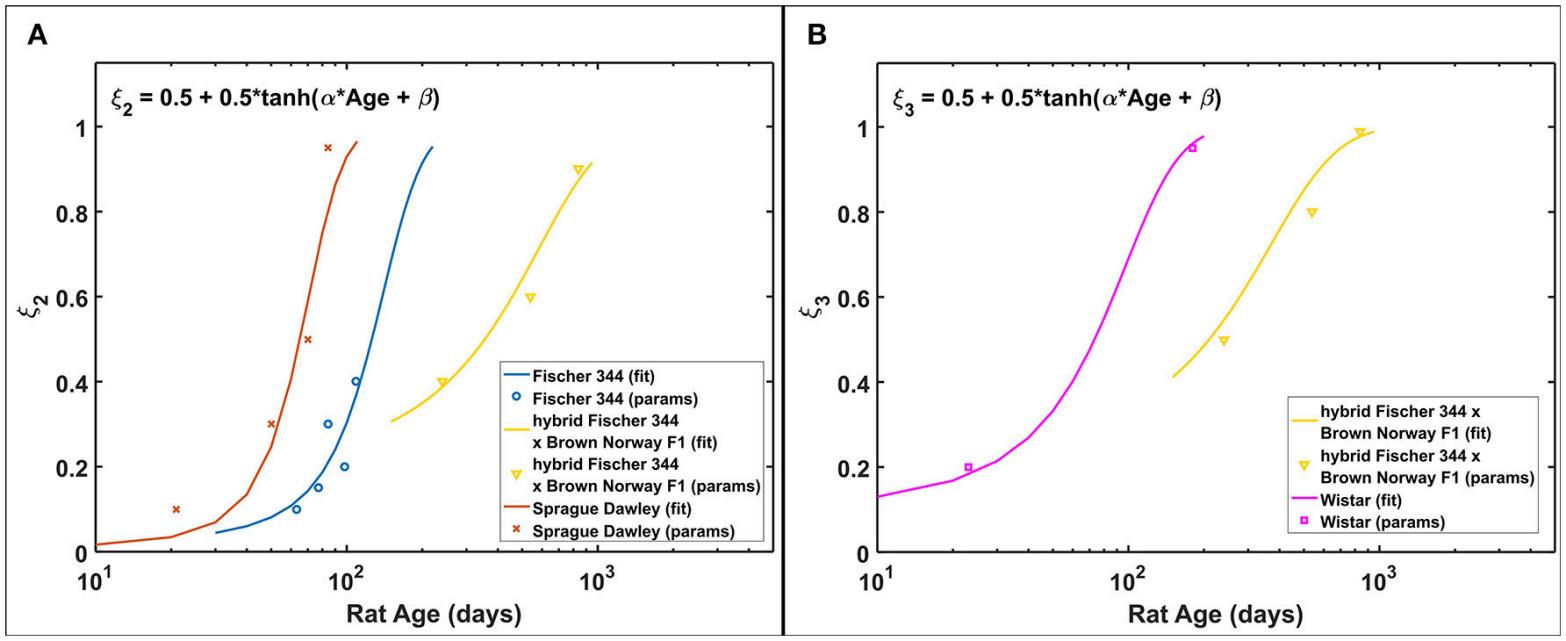
Rat strain and age dependence of repair related parameters of hippocampal neurogenesis after irradiation. **(A)** NB fraction of repairable weakly damaged cells (ξ_2_). **(B)** ImN fraction of repairable weakly damaged cells (ξ_3_).

Based on the estimated radiation damage and repair related parameters presented in Table 2, we formulate a function to describe the fraction of repairable weakly damaged NB (ξ_2_) and ImN (ξ_3_) cells as shown in Figure 7, where coefficients of the given equation are found in Table 4. Eventually, this equation can be used to generate predictions on the dependence of repair related parameters of hippocampal neurogenesis after radiation treatment on the age and strain of rat models. Before adulthood or 6 months of age (Sengupta, 2013), Sprague Dawley and Fischer 344 rat strains are predicted to have the majority of their weakly damaged NB cells to be repairable while approximately only half of the population are repairable for hybrid Fischer 344 × Brown Norway F1 rats. Likewise, most of the weakly damaged ImN cells are repairable in Wistar rats by the time of adulthood and only half in hybrid Fischer 344 × Brown Norway F1 rats, with fraction of repairable weakly damaged NB and ImN cells increases further with age.

**Table 4:** Fitting coefficients to repair related model parameters.

**Rat strain**	**α (day^**−1**^) [95% CI]**	**β [95% CI]**
**NB FRACTION OF REPAIRABLE WEAKLY DAMAGED CELLS (ξ_2_)**		
Fischer 344 rat	0.016 [−0.006, 0.038]	−2.02 [−4.14, 0.11]
hybrid Fischer 344 × Brown Norway F1	0.002 [−0.005, 0.008]	−0.71 [−3.89, 2.47]
Sprague Dawley rat	0.038 [−0.026, 0.10]	−2.41 [−6.67, 1.85]
**ImN FRACTION OF REPAIRABLE WEAKLY DAMAGED CELLS (ξ_3_)**		
hybrid Fischer 344 × Brown Norway F1	0.003 [−0.003, 0.008]	−0.63 [−2.75, 1.49]
Wistar	0.015 [−0.018, 0.047]	−1.1 [−2.53, 0.33]

## Discussion

In this work, we have expanded our previous mathematical model of radiation-induced changes to neurogenesis of mouse models to delineate and make predictions of the observed neurogenesis alterations in different age and strain of rats. Cell kinetics are modeled using a system of non-linear differential equations, which describe key cell populations involved in hippocampal neurogenesis, which includes neural stem cells (NSC), neuronal progenitor cells or neuroblasts (NB), immature neurons (ImN), and glioblasts (GB). In Figure 2, modeling results in comparison to experimental data in male rats show that genetic background of various rat strains influences the rate of neurogenesis. For instance, the hybrid Fischer 344 × Brown Norway F1 rat strain has relatively slower neurogenesis compared to Wistar rat, which might be partially due to their differences in life span, food consumption and growth characteristics where the former has been known to live longer (Turturro et al., 1999) that makes it ideal for age-related studies as recommended by the National Institute on Aging. Regardless, our modeling results are consistent with the experimental data in literature about genetic influences on different aspects of neurogenesis in adult mice (Kempermann et al., 1997; Kim et al., 2017). More analysis about the effect of genetic background on neurogenesis is found in succeeding discussion. Furthermore, our modeling results closely match data on the effects of radiation on hippocampal neurogenesis of various age and strain of rat models from 12 studies as outlined in Supplementary Table 3. Our model emulates the published experimental data that involve descriptions of proliferating cell marker Ki67 for NB, immature neuron marker Dcx for ImN, ratio of activated microglia marker CD68 over total microglia marker Iba-1 for increased microglial activation (μ), and co-labeling of proliferation marker BrDU and mature neuron marker NeuN for neurogenic fate (Δ). Similar to our earlier model, we verify for all rat strains being considered that the estimated NSC differentiation to NB and GB parameter (d_1_) would be approximately an order of magnitude lower than the maximum NSC proliferation (Ψ_1_) to achieve a stable equilibrium state and avoid a state of complete extinction.

The radiation response of neuronal progenitor cells shows significant reduction in number of proliferating cells and immature neurons at 24 h post-irradiation, while cell proliferation increases between 1 and 7 days after exposure (Fike et al., 2007). Early cell loss after irradiation was attributed to programmed cell death (apoptosis) that peaks at 6 h and goes to completion at 24–48 h after exposure (Bellinoza et al., 1996). Furthermore, most of the cells undergoing apoptosis were proliferating cells especially at lower doses (Shinohara et al., 1997). As shown in Figure 3C, our model elucidates this transient decrease in the Ki67 marker that occurs at 1–2 days post-irradiation and then an increase in proliferation that peaks at 7 days after radiation exposure. Aside from radiation treatment, increased proliferation of neuronal progenitor cells is recognized to be caused by several CNS injuries, such as brain ischemia (Cho and Yun Kim, 2010; Tobin et al., 2014), traumatic brain injury (Cho and Yun Kim, 2010), epileptic seizures (Cho and Yun Kim, 2010), and some pharmacological manipulations, for instance, antidepressant fluoxetine (Cho and Yun Kim, 2010) and binge-like alcohol exposure (Geil Nickell et al., 2017). Elevated proliferation is observed to peak at around 7 days after brain insult then quickly returns to baseline. The mechanism of increased number of produced neuroblasts in response to brain insults and the ultimate fate of these newly born cells still remains unknown (Bonfanti, 2016).

Radiation-induced neurogenesis impairment is often accompanied with neuroinflammation, specifically the newly born or activation of microglial cells. As the primary resident immune cells of the CNS, microglia constantly monitor the brain environment and act as host defense in response to stimuli by releasing pro-inflammatory molecules during their activated state. Besides the increase in basal activation of microglial cells with aging (Ogura et al., 1994; Wong, 2013), a more dysregulated response of microglial cells to CNS perturbations is anticipated with old age, producing an excessive and prolonged activation of microglia (Wong, 2013). As shown in Figures 5C,D, microglial activation elevated with increasing age of unirradiated hybrid Fischer 44 × Brown Norway F1 rats and greater and prolonged microglial activation in Wistar rats were observed in experiments and recognized by our model. Moreover, studies of Monje and co-workers (Monje et al., 2002, 2003) suggested an important role of activated microglia in reduced NSC proliferation and a possible shift of NSC proliferation from neurogenesis to gliogenesis. However, a reduced role of activated microglia and no concurrent shift leading to gliogenesis has been observed in other studies with X-rays and low LET proton irradiation (Naylor et al., 2008; Liao et al., 2013; Sweet et al., 2014). Studies of Sweet and co-workers has suggested that differences may occur due to the time and frequency of BrDU proliferation labeling, including the possibility that pools of NSC may be dividing at different times. In any case, a role of activated microglia and neurogenic fate (shift to gliogenesis) is included in the negative feedback regulation on proliferation dynamics of neural stem and progenitor cells. This approach is based on Smirnova's modeling that has been used to describe the dynamics of blood and skin systems after radiation exposure (Smirnova, 2011; Smirnova et al., 2014a,b). Since its first description in hematopoietic systems, maintaining tissue homeostasis through regulation of stem cells by feedback signals produced by daughter cells has been recognized and well-studied (Rao Tata and Rajagopal, 2016). Regulatory mechanisms on neural stem cells might not be as definitive compared to hematopoiesis, however, several mechanisms of proliferative feedback on neural stem cells have been reported to depend on specific molecular pathways and neurotransmitters, such as glutamate and GABA (Song et al., 2012; Faigle and Song, 2013; Pallotto and Deprez, 2014). The current feedback regulation presented as a function of cell populations and fraction of increased activated microglia can be modified to describe other proliferative feedback mechanisms once experimental data describing specific regulatory mechanisms have been made available. Furthermore, the delayed response of activated microglia recognized in C57BL/6 mouse is not consistent with some rat strains. For instance, in Wistar rats, transient activation of multiple inflammatory mechanisms (CCL2, Gro/KC, and IL-1α) in the acute phase (6 h post-IR) and activation of astrocytes in the subacute phase (7 days post-IR) were observed (Kalm et al., 2009). While there is limited experimental data available, the parametric description presented in our model is adequate to delineate radiation-induced microglial activation recognized in rodent models. However, additional investigation will be needed to improve model predictions.

In Figure 6, modeling results show that acute radiation exposure results in more damage compared to fractionated exposures. Gaber et al. (2003) have attributed this to the expression of intercellular adhesion molecule (ICAM-1) and tumor necrosis factor-α (TNF-α) where rapid molecular response of the brain is observed for single-dose irradiation in contrast to a slow reaction for fractionated irradiation in mouse brain. However, caution must be made in comparing damage of single (acute) radiation exposure to fractionated exposures. Cell damage due to fractionated exposures is dependent on fractionation radiation dose and schedule and on the repair mechanism of the rodent model. Thus, it is not simple to directly compare the damage of single dose to fractionated exposure of one condition in a rodent model to another condition of a different rodent. On the other hand, increased activation of microglial cells is greater in acute than in fractionated radiation exposures, while a shift toward gliogenesis is favored by acute exposures compared to fractionated treatment.

Basal strain-dependent differences in hippocampal neurogenesis is generally associated with genotypes and phenotypes from the rodent's genetic background (Kempermann et al., 1997; Kim et al., 2017). Several possible mechanisms could account for the observed strain-related differences in postnatally generated granule cell numbers, such as differences in cell division, differentiation or death. Besides genetic factors, hormonal and environmental stimuli are crucial determinants of the differences in neurogenesis. For instance, diversity in the number of α-adrenergic receptors and neurotransmitters were considered to influence differences in the number of dentate granule cells in different strains of rat (Boss et al., 1985). Likewise, brain-derived neurotrophic factor (BDNF), which is a known regulator of neurogenesis, appears to be dependent on genetic background of rats (Johnson and Mitchell, 2003). Variations in genetic background might be one of the factors that affect the contrasting results presented in the recent controversy as to whether human neurogenesis persists throughout life (Boldrini et al., 2018; Sorrells et al., 2018). Even though rats are known to be more genetically diverse than humans (Rat Genome Sequencing Project Consortium, 2004), species differences and other considerations are important in the investigation of adult neurogenesis (Kempermann et al., 2018). On the other hand, these distinction in genetic and environmental backgrounds among rodent species and strains may also influence the sensitivity of hippocampal neurogenesis to radiation exposures. However, it cannot be ruled out that inter-laboratory differences in methods, such as differences in the time-courses and frequency of BrDU proliferation labeling, could lead to a portion of the variations described (Supplementary Table 4 compares labeling approaches in the experiments noted).

Our model predicts that characteristic dose of immature neurons (D_03_) is, in most cases, lower in rats than in C57BL/6 mice. Our previous model indicates D_03_ = 7.5 Gy for C57BL/6 mouse, while D_03_ = 3.1 ± 1.4 Gy for Fischer 344, Sprague Dawley, and Wistar rats (excluding hybrid Fischer 344 × Brown Norway F1 where D_03_ = 10 Gy). It has been reported that maturation of immature neurons in mice (C57BL/6 and CD1) occur within a 3 weeks delay compared to rats (Sprague Dawley and Long Evans) (Snyder et al., 2009). Faster neuron maturation in rats compared to mice makes immature neurons of the former more susceptible to radiation damage, hence, lower D_03_ value. Moreover, as radiation damages represented by characteristic doses (D_02_ and D_03_) depends on rat strain, repair related parameters of hippocampal neurogenesis (ξ_2_ and ξ_3_) are simulated to be influenced by both age and strain of rats. Repair and misrepair rates for weakly damaged cells are not well determined in experiments, and we would need variable dose-rate and additional dose fractionation investigations for different age and strain of rats to improve estimates of these parameters and to better analyze our model assumptions. The model described in Figure 7 with values in Table 4 suggest there is a shift from largely exponential cell survival curves with little sub-lethal damage repair at younger ages to a large increase in sub-lethal damage repair at older ages. This shift is in-line with the large background losses of neurons that occur at younger ages, which suggests repair mechanisms operate to remove damaged cells to a larger extent when there are an excessive number of neurons present. While the equations describing the fraction of repairable weakly damaged cells are presently speculative, this contributes to understanding the age and strain dependence of radiation-induced hippocampal neurogenesis impairment and may be valuable in guiding future experimentation.

Several mathematical modeling studies of neurogenesis have been published (Ziebell et al., 2014, 2018; Beccari et al., 2017; Li et al., 2017; Marie et al., 2018) and these models have considered comprehensive analysis of neural stem and progenitor cells behaviors. In our model, we have simplified the neuroprogenitor behavior by using an “end-point approach” in which all neuronal progenitor cell populations (types 2a, 2b, and 3) are analyzed together. This approach works well and sufficient in emulating the existing experimental data studying the effects of radiation on hippocampal neurogenesis. For future work, we plan on including a more detailed analysis on proliferation rate of different types of neuroprogenitor cells to better understand differences in various experiments on neuroprogenitor cells proliferation, dose dependence of the inflammatory responses and microglial activation along with the possible shift from neurogenesis to gliogenesis. Also, even though a clinically relevant radiation dose did not produce significant damage on vascular niche, it would be interesting to model the experimental data observed that irradiation changes the neurogenic-angiogenic relationship by altering the proliferative neuronal precursor cell clustering in close proximity to the vascular niche, such that radiation induced small clustering and longer distance from microvasculature (Palmer et al., 2000; Monje et al., 2002). Furthermore, since it is more likely that multiple mechanisms are in play for radiation-induced hippocampal neurogenesis damages and its correlation to cognitive dysfunction, alternative approaches to both experimental and modeling design (Lazic, 2011, 2012; Nakagawa and Hauber, 2011; Jessberger and Gage, 2014) should be considered to elucidate causative mechanisms and to translate studies in rodent and other animal models to human physiology that would be beneficial in optimizing radiation therapy in cancer patients. Finally a cross-species description of blood cell kinetics in radiation exposures has been previously developed (Hu and Cucinotta, 2011), and a similar approach could be investigated for human brain irradiations using our approach.

## Author Contributions

EC, SK, and FC analyzed data, formulated the model, and performed simulations. EC and FC wrote the manuscript. All authors reviewed the manuscript.

### Conflict of Interest Statement

The authors declare that the research was conducted in the absence of any commercial or financial relationships that could be construed as a potential conflict of interest.
