# Supplementary material for: Modeling Reveals the Dependence of Hippocampal Neurogenesis Radiosensitivity on Age and Strain of Rats
Source: Front Neurosci. 2018 Dec 20;12:980. doi: 10.3389/fnins.2018.00980 (PMC6306485; doi:10.3389/fnins.2018.00980)
Supplement: Supplementary file 1 [file Table_1.DOCX]

Supplementary Material

Modeling reveals the dependence of hippocampal neurogenesis radiosensitivity on age and strain of rats

Eliedonna Cacao, Sidath Kapukotuwa and Francis A. Cucinotta*

***Correspondence:** Francis A. Cucinotta: francis.cucinotta@unlv.edu

**Supplementary Table 1. Hippocampal neurogenesis parameters**

**that are independent of rat strain and age**

| ***UNIRRADIATED/CONTROL*** | |
| --- | --- |
| ***Parameters (unit)*** | ***Value*** |
| Ψ_1_ (day^-1^) | 5 x 10^-1^ |
| Ψ_2_ | 5 x 10^2^ |
| θ_1_ | 5 x 10^-3^ |
| θ_2_ | 5 x 10^-2^ |
| θ_3_ | 5 x 10^-3^ |
| a_4_ (day^-1^) | 1 x 10^-2^ |
| x_a_ | 0.60 |
| x_b_ | 0.40 |
| ***IRRADIATED*** | |
| ***Parameters (unit)*** | ***Value*** |
| ν_2_ (day^-1^) | 14 |
| ν_3_ (day^-1^) | 1.4 |
| ν_5_ (day^-1^) | 2.1 |
| ω | 0.40 |
| θ_mg_ | 50 |
| Φ | 1 |
| Γ | 1.05 |
| ***Neurogenic fate (Δ) related parameters*** | |
| A_0_ | - 6 x 10^-2^ |
| A_1_ (Gy) | 9 |
| B_0_ | - 9.6 x 10^-3^ |
| B_1_ (day^-1^) | 1 x 10^-5^ |
| C (day^-2^) | 1 x 10^-6^ |
| λ (day^-1^) | 4 x 10^-2^ |

**Supplementary Table 2. Summary of hippocampal neurogenesis experimental data on different rat strains and their corresponding references**

| **Rat strain** | **Age (days)** | **References** |
| --- | --- | --- |
| Fischer 344 | 120 - 720 | Amrein, I., et. al. 2011  Rao, M., et. al. 2005  Rao, M., et. al. 2006 |
| Hybrid Fischer 344 x Brown Norway F1 | 240 - 903 | Schindler, M.K., et. al. 2008 |
| Sprague Dawley | 35 - 200 | Lazic, S.E. 2012  Epp, J.R., et. al. 2009 |
| Wistar | 9 - 94 | Amrein, I., et. al. 2011  Fukuda, A., et. al. 2005 |
| Long Evans | 72 - 335 | Merkley, C.M., et. al. 2014 |

**Supplementary Table 3. Summary of experimental data of radiation-induced damages of hippocampal neurogenesis on different rat strains and their corresponding references**

| **Rodent model** | **Age (days)** | **Radiation dose range and rate, type and exposure scheme** | **References** |
| --- | --- | --- | --- |
| Fischer 344 | 63 | 1 – 30 Gy, 1.75 Gy/min,  X-rays (acute) | Tada, E., et. al. 2000 |
|  | 77 | 11 – 40 Gy, 4 Gy/min,  ^137^Cs γ-rays  (acute and fractionated) | Greene-Schloesser, D. et. al. 2014 |
|  | 84 | 10 Gy, 3.2 Gy.min,  ^137^Cs γ-rays (acute) | Jenrow, K.A., et. al. 2013 |
|  | 98 | 7.5 – 15 Gy, 0.089 Gy/min,  X-rays (acute) | Otsuka, S., et. al. 2006 |
|  | 109 | 10 Gy, 3 Gy/min,  X-rays (acute) | Peibner, W., et. al. 1999 |
| Hybrid Fischer 344 x Brown Norway F1 | 77 | 40 Gy, 4.41 Gy/min,  ^137^Cs γ-rays (fractionated) | Conner, K.R., et. al. 2010 |
|  | 240 | 10 Gy, 4.23 Gy/min,  ^137^Cs γ-rays (acute) | Schindler, M.K., et. al. 2008 |
|  | 540 |  |  |
|  | 840 |  |  |
| Sprague Dawley | 21 | 0.3 – 10 Gy, 0.6 Gy/min,  X-rays (acute) | Achanta, P., et. al. 2009 |
|  | 50 |  |  |
|  | 70 |  |  |
|  | 84 | 1 – 10 Gy, 0.6 Gy/min,  X-rays (acute) | Tan, Y.F., et.al. 2011 |
| Wistar | 9 | 8 Gy, 2.1 Gy/min,  X-rays (acute) | Kalm, M., et. al. 2009 |
|  | 21 |  |  |
|  | 9 | 8 Gy, 2.3 Gy/min,  X-rays (acute) | Fukuda, A., et. al. 2005 |
|  | 23 |  |  |
|  | 9 | 6 Gy, 2.1 Gy/min,  X-rays (acute) | Blomstrand, M., et. al. 2014 |
|  | 180 |  |  |

**Supplementary Table 4. Summary of labeling methods used in hippocampal neurogenesis experimental data of different rat strains and their corresponding references**

| **Fischer 344** |
| --- |
| - ***Proliferation***: detected with monoclonal antibody against Ki67, labeled with avidin-biotin-peroxidase and visualized with vector grey or DAB chromogen using optical microscopy (Amrein, I., et. al. 2011; Rao, M., et. al. 2005; Rao, M., et. al. 2006; Otsuka, S., et. al. 2006; Peibner, W., et. al. 1999) or labeled with secondary antibody- fluorophore conjugate and visualized using fluorescence microscopy (Jenrow, K.A., et. al. 2013). - ***Immature neurons***: detected with polyclonal antibody against Dcx, labeled with avidin-biotin-peroxidase and visualized with vector grey or DAB chromogen using optical microscopy (Amrein, I., et. al. 2011; Rao, M., et. al. 2005; Rao, M., et. al. 2006; Otsuka, S., et. al. 2006; Peibner, W., et. al. 1999) or labeled with secondary antibody- fluorophore conjugate and visualized using fluorescence microscopy (Jenrow, K.A., et. al. 2013). - ***Apoptosis***: detected and labeled with TUNEL staining and visualized with DAB chromogen using optical microscopy (Tada, E., et. al. 2000; Peibner, W., et. al. 1999). - ***Total and Activated microglia***: detected with polyclonal antibody against Iba-1 (total microglia) or monoclonal antibody against CD68/ED-1 (activated microglia), labeled with avidin-biotin peroxidase and visualized with nickel enhanced DAB chromogen using optical microscopy (Greene-Schloesser, D. et. al. 2014). - ***Neurogenic fate (surviving newborn neurons)***: intraperitoneal (IP) injection of 50 mg/kg BrdU for 7 days starting 1 month after irradiation); rats were euthanized at 2 months postIR; detection using monoclonal antibody against BrdU and NeuN, double labeling with secondary antibody-Cy3 fluorophore conjugate for BrdU and secondary antibody-Alexa 488 fluorophore conjugate for NeuN, and visualization using fluorescence microscopy (Greene-Schloesser, D. et. al. 2014). |
| **Hybrid Fischer 344 x Brown Norway F1** |
| - ***Proliferation***: detected with monoclonal antibody against Ki67, labeled with avidin-biotin-peroxidase and visualized with Vector SG or DAB chromogen using optical microscopy (Schindler, M.K., et. al. 2008; Conner, K.R., et. al. 2010). - ***Immature neurons***: detected using polyclonal antibody against Dcx, labeled with avidin-biotin peroxidase and visualized with DAB chromogen using optical microscopy (Schindler, M.K., et. al. 2008) or or labeled with secondary antibody- Cy5 fluorophore conjugate and visualized using fluorescence microscopy (Conner, K.R., et. al. 2010). - ***Total and Activated microglia***: detected with polyclonal antibody against Iba-1 (total microglia) or monoclonal antibody against CD68/ED-1 (activated microglia), labeled with avidin-biotin peroxidase and visualized with DAB or nickel-enhanced DAB chromogen using optical microscopy (Schindler, M.K., et. al. 2008; Conner, K.R., et. al. 2010). |
| **Sprague Dawley** |
| - ***Proliferation***: detected with monoclonal antibody against Ki67, labeled with secondary antibody-Alexa 488 or 546 or 594 fluorophore conjugate and visualized using fluorescence microscopy (Epp, J.R., et. al. 2009; Achanta, P., et. al. 2009; Tan, Y.F., et. al. 2011). - ***Immature neurons***: detected with monoclonal antibody against Dcx, labeled with secondary antibody-Alexa 488 or 594 fluorophore conjugate and visualized using fluorescence microscopy (Epp, J.R., et. al. 2009; Tan, Y.F., et. al. 2011). - ***Neurogenic fate (surviving newborn neurons)***: three daily intraperitoneal (IP) injection of 200 mg/kg BrdU administered a week before irradiation); rats were euthanized at 3 months postIR; detection using monoclonal antibody against BrdU and NeuN, double labeling with secondary antibody-Alexa 488 fluorophore conjugate for BrdU and secondary antibody-Alexa 594 fluorophore conjugate for NeuN, and visualization using fluorescence microscopy (Achanta, P., et. al. 2009). |
| **Wistar** |
| - ***Neural stem cells***: detected with monoclonal antibody against nestin, labeled with avidin-biotin-peroxidase and visualized with DAB chromogen using optical microscopy (Amrein, I., et. al. 2011; Fukuda, A., et. al. 2005). - ***Immature neurons***: detected with monoclonal antibody against Dcx, labeled with avidin-biotin-peroxidase and visualized with DAB chromogen using optical microscopy (Amrein, I., et. al. 2011; Fukuda, A., et. al. 2005). - ***Total and Activated microglia***: detected with polyclonal antibody against Iba-1 (total microglia) or monoclonal antibody against CD68/ED-1 (activated microglia), labeled with secondary antibody-fluorophore conjugate and visualized using fluorescence microscopy (Kalm, M., et. al. 2009; Blomstrand, M., et. al. 2014). |
| **Long Evans** |
| - ***Proliferation***: detected with monoclonal antibody against Ki67, labeled with secondary antibody-Alexa 568 fluorophore conjugate and visualized using fluorescence microscopy. - ***Immature neurons***: detected with monoclonal antibody against Dcx, labeled with secondary antibody-Alexa 488 fluorophore conjugate and visualized using fluorescence microscopy. |

**REFERENCES**

1. Amrein, I., Isler, K. and Lipp, H-P. (2011). Comparing adult hippocampal neurogenesis in mammalian species and orders: influence of chronological age and life history stage. Eur. J. Neurosci. 34(6): 978 – 987. Doi: 10.1111/j.1460-9568.2011.07804.x.
2. Rao, M.S., Hattiangady, B., Abdel-Rahman, A., Stanley, D.P. and Shetty, A.K. (2005). Newly born cells in the ageing dentate gyrus display normal migration, survival and neuronal fate choice but endure retarded early maturation. Eur. J. Neurosci. 21: 464 – 476. Doi: 10.1111/j.1460-9568.2005.03853.x
3. Rao, M.S., Hattiangady, B. abd Shetty, A.K. (2006). The window and mechanisms of major age-related decline in the production of new neurons within the dentate gyrus of the hippocampus. Aging Cell. 5: 545 – 558. Doi: 10.1111/j.1474-9726.2006.00243.x.
4. Schindler, M.K., Elizabeth Forbes, M., Robbins, M.E. and Riddle, D.R. (2008). Aging-dependent changes in the radiation response of the adult rat brain. Int. J. Radiat. Oncol. Biol. Phys. 70(3): 826 – 834. doi: 10.1016/j.ijrobp.2007.10.054.
5. Lazic, S.E. (2012). Modeling hippocampal neurogenesis across the lifespan in seven species. Neurobiol. Aging. 33(8): 1664 – 1671. doi: 10.1016/j.neurobiolaging.2011.03.008.
6. Epp, J.R. Barker, J.M. and Galea, L.A.M. (2009). Running wild: neurogenesis in the hippocampus across the lifespan in wild and laboratory-bred Norway rats. Hippocampus. 19: 1034 – 1043. doi: 10.1002/hipo.20546.
7. Fukuda, A., Fukuda, H., Swanpalmer, J., Hertzman, S., Lannering, B., Marky, I., et. al. (2005). Age-dependent sensitivity of the developing brain to irradiation is correlated with the number and vulnerability of progenitor cells. J Neurochem. 92(3): 569 – 584. doi: 10.1111/j.1471-4159.2004.02894.x.
8. Merkley, C.M., Jian, C., Mosa, A., Yao-Fang, T. and Martin Wojtowicz, J. (2014). Homeostatic regulation of adult hippocampal neurogenesis in aging rats: long-term effects of early exercise. Frontiers in Neuroscience. 8(174): 1 – 12. doi: 10.3389/fnins.2014.00174.
9. Tada, E., Parent, J.M., Lowenstein, D.H. and FIke, J.R. (2000). X-irradiation causes a prolonged reduction in cell proliferation in the dentate gyrus of adult rats. Neuroscience. 99(1): 33 – 41. doi: 10.1016/S0306-4522(00)00151-2.
10. Greene-Schloesser, D.M., Kooshki, M., Payne, V., D’Agostino, Jr., R.N., Wheeler, K.T., Metheny-Barlow, L.J. et. al. (2014). Cellular response of the rat brain to single doses of ^137^Cs γ-rays does not predictex its response to prolonged “biologically equivalent” fractionated doses. Int. J. Radiat. Biol. 90(9): 790 – 798. doi: 10.3109/09553002.2014.933915.
11. Jenrow, K.A., Brown, S.L., Lapanowski, K., Naei, H., Kolozsvary, A. and Kim, J.H. (2013). Selective inhibition of microglia-mediated neuroinflammation mitigates radiation-induced cognitive impairment. Radiat. Res. 179: 549 – 556. doi: 10.1667/RR3026.1.
12. Otsuka, S., Coderre, J.A., Micca, P.L., Morris, G.M., Hopewell, J.W., Rola, R., et. al. (2006). Depletion of neural precursor cells after local brain irradiation is due to radiation dose to the parenchyma, not the vasculature. Radiat. Res. 165: 582 – 591. doi: 10.1667/RR3539.1.
13. Peibner, W., Kocher, M., Treuer, H. and Gillardon, F. (1999). Ionizing radiation-induced apoptosis of proliferating stem cells in the dentate gyrus of the adult rat hippocampus. Mol. Brain Res. 71(1): 61 – 68. doi: 10.1016/S0169-328X(99)00170-9.
14. Conner, K.R., Payne, V.S., Elizabeth-Forbes, M., Robbins, M.E. and Riddle, D.R. (2010). Effects of the AT_1_ receptor antagonist L-158,809 on miroglia and neurogenesis after fractionated whole-brain irradiation. Radiat. Res. 173(1): 49 – 61. doi: 10.1667/RR1821.1.
15. Achanta, P., Fuss, M. and Martinez, Jr., J.L. (2009). Ionizing radiation impairs the formation of trace fear memories and reduces hippocampal neurogenesis. Behav. Neurosci. 123(5): 1036 – 1045. doi: 10.1037/a0016870.
16. Tan, Y.F., Rosenzweig, S., Wojtowicz, J.M. (2011). Depletion of new neurons by image guided irradiation. Front. Neurosci. 5: 59. doi: 10.3389/fnins.2011.00059.
17. Kalm, M., Lannering, B., Bjork-Eriksson, T. and Blomgren, K. (2009). Irradiation-induced loss of micoglia in the young brain. J. Neuroimmunol. 206(1-2): 70 – 75. doi: 10.1016/j.jneuroim.2008.11.002.
18. Blomstrand, M., Kalm, M., Grander, R., Bjork-Eriksson, T., and Blomgren, K. (2014). Different reactions to irradiation in the juvenile and adult hippocampus. Int. J. Radiat. Biol. 90(9): 807 – 815. doi: 10.3109/09553002.2014.942015.
